# Blood lactate levels are associated with an increased risk of metabolic dysfunction-associated fatty liver disease in type 2 diabetes: a real-world study

**DOI:** 10.3389/fendo.2023.1133991

**Published:** 2023-05-08

**Authors:** Yi-Lin Ma, Jiang-Feng Ke, Jun-Wei Wang, Yu-Jie Wang, Man-Rong Xu, Lian-Xi Li

**Affiliations:** ^1^ Department of Endocrinology and Metabolism, Shanghai Sixth People's Hospital Affiliated to Shanghai Jiao Tong University School of Medicine, Shanghai Clinical Center for Diabetes, Shanghai Diabetes Institute, Shanghai Key Laboratory of Diabetes Mellitus, Shanghai Key Clinical Center for Metabolic Disease, Shanghai, China; ^2^ Department of Radiation Oncology, Clinical Oncology School of Fujian Medical University, Fujian Cancer Hospital, Fuzhou, China

**Keywords:** lactate, metabolic dysfunction-associated fatty liver disease, type 2 diabetes mellitus, insulin resistance, HOMA2-IR

## Abstract

**Aim:**

To investigate the association between blood lactate levels and metabolic dysfunction-associated fatty liver disease (MAFLD) in type 2 diabetes mellitus (T2DM).

**Methods:**

4628 Chinese T2DM patients were divided into quartiles according to blood lactate levels in this real-world study. Abdominal ultrasonography was used to diagnosis MAFLD. The associations of blood lactate levels and quartiles with MAFLD were analyzed by logistic regression.

**Results:**

There were a significantly increased trend in both MAFLD prevalence (28.9%, 36.5%, 43.5%, and 54.7%) and HOMA2-IR value (1.31(0.80-2.03), 1.44(0.87-2.20), 1.59(0.99-2.36), 1.82(1.15-2.59)) across the blood lactate quartiles in T2DM patients after adjustment for age, sex, diabetic duration, and metformin use (all *p*<0.001 for trend). After correcting for other confounding factors, not only increased blood lactate levels were obviously associated with MAFLD presence in the patients with (OR=1.378, 95%CI: 1.210-1.569, *p*<0.001) and without taking metformin (OR=1.181, 95%CI: 1.010-1.381, *p*=0.037), but also blood lactate quartiles were independently correlated to the increased risk of MAFLD in T2DM patients (*p*<0.001 for trend). Compared with the subjects in the lowest blood lactate quartiles, the risk of MAFLD increased to 1.436-, 1.473-, and 2.055-fold, respectively, in those from the second to the highest lactate quartiles.

**Conclusions:**

The blood lactate levels in T2DM subjects were independently associated with an increased risk of MAFLD, which was not affected by metformin-taking and might closely related to insulin resistance. Blood lactate levels might be used as a practical indicator for assessing the risk of MAFLD in T2DM patients.

## Introduction

Metabolic dysfunction-associated fatty liver disease (MAFLD), formerly known as non-alcoholic fatty liver disease (NAFLD), is a new and accurate definition established by international expert consensus in 2020. MAFLD includes a range of liver pathological processes from hepatic steatosis to liver inflammation, fibrosis, cirrhosis, and finally to hepatocellular carcinoma. The diagnosis of MAFLD was based on histology, imaging, or blood biomarker evidence of hepatic steatosis and on the co-existence of overweight/obesity, or type 2 diabetes mellitus (T2DM) or other metabolic abnormalities (1). Compared with NAFLD, the MAFLD population includes subjects with alcohol consumption and the new definition of MAFLD focuses more on systemic metabolic changes rather than on individual organ systems. Therefore, the MAFLD criteria identifies patients at high risk for the progression of liver fibrosis and for other significant complications such as atherosclerotic cardiovascular disease (2, 3).

MAFLD is the leading cause of chronic liver disease worldwide with a 39.22% overall prevalence in general population (4). More notably, T2DM patients have a higher risk of developing MAFLD than general population (5). Recent meta-analysis studies reported that the estimated prevalence of global MAFLD was about 55.48% in T2DM patients, even with regional prevalence of 51.83% in the mainland China (6, 7). Considering the high prevalence and great harm of MAFLD, and the important role of metabolic disorders in the pathogenesis of MAFLD, it is crucial to elucidate the mechanisms of metabolic disturbance of hepatocyte in MAFLD. It’s well established that several metabolic pathways including enhanced glycolysis and reduced mitochondrial respiration were significantly altered in hepatocytes in MAFLD (8). Therefore, as the consequence of glycolysis under hypoxic or anaerobic conditions, circulating lactate is considered as the main source of carbon for the tricarboxylic acid cycle, which provides energy for tissues such as skeletal muscle and brain (9, 10).

Currently, blood lactate was regarded as a reflection of hepatocellular failure, several studies have confirmed that MAFLD is characterized by the upregulation of lactate levels (11–13). For example, Toye and colleagues observed that plasma lactate levels were significantly increased in high-fat-fed mice with NAFLD (11). Likewise, Li et al. selected serum lactate as a potential biomarker for diagnosis of NAFLD stages based on comparative analysis of differential serum metabolites in normal and NAFLD mice at different stages of NAFLD, and found that serum lactate levels were also significantly elevated in NAFLD mice established by feeding MCD diet beyond two weeks (12). Interestingly, this phenomenon was only present in NAFLD patients with steatosis alone, but not in steatosis patients with necro-inflammatory disease and NASH patients (12).

Nevertheless, studies on the association between lactate and MAFLD have mostly focused on animal experiments, few in the human population (11–16). For example, according to plasma metabolomic analysis, a small sample study in non-diabetic subjects reported that compared with healthy controls, plasma lactate levels were higher in both non-diabetic patients with histologically confirmed hepatic steatosis and steatohepatitis (13). Similarly, the higher fasting lactate levels were found in the cirrhotic patients compared with the controls (16). Thus, the relationship between blood lactate and MAFLD remained to be explored, especially in T2DM populations.

The present study aimed to investigate the correlation between blood lactate levels and MAFLD with a relatively large sample size in hospitalized T2DM subjects, and to further determine whether blood lactate could be used as an early biomarker to evaluate the risk of MAFLD.

## Materials and methods

### Study population and design

This real-world, cross-sectional study was authorized by the ethics committee of Shanghai Sixth People’s Hospital Affiliated to Shanghai Jiao Tong University School of Medicine (approved number: 2018-KY-018(K)) and conformed to the ethical guidelines of the Declaration of Helsinki. This study consecutively recruited T2DM patients hospitalized in the Department of Endocrinology and Metabolism from January 2003 to August 2009. The written informed consent was obtained from each participant. Some participants were excluded for the following reasons: without the results of blood lactate and abdominal ultrasonography; hepatic impairment caused by drugs, viral hepatitis, and other reasons excluding alcohol consumption; with acute diabetic complications such as ketoacidosis; increased lactate levels caused by diseases such as acute severe asthma, severe heart failure and malignancy or drugs such as acetaminophen but excluding metformin (17, 18). Ultimately, a total of 4628 T2DM subjects were enrolled in the present study and then divided into four groups according to blood lactate quartiles.

### Data collection

Basic medical information was obtained from interviews with subjects including age, sex, duration of diabetes (DD), smoking status, alcohol use, hypertension, and medications including insulin or insulin analogs (IIAs), lipid-lowering drugs (LLDs), metformin, and insulin sensitizers.

Physical measurements included height, weight, waist circumference (WC), hip circumference, systolic blood pressure (SBP), diastolic blood pressure (DBP). The calculation of waist-to-hip ratio (WHR) and body mass index (BMI) were referred to our recent studies (19, 20).

The laboratory measurements and blood sample collection had been mentioned in detail in our recent study (21). The laboratory tests in this study included fasting plasma glucose (FPG), 2-hour postprandial plasma glucose (2-h PPG), glycosylated hemoglobin A1c (HbA1c), fasting C-peptide (FCP), postprandial 2-hour postprandial C-peptide (2-h PCP), the levels of triglyceride (TG), total cholesterol (TC), high-density lipoprotein cholesterol (HDL-C), low-density lipoprotein cholesterol (LDL-C), creatinine (Cr), serum uric acid (SUA), 24-hour urinary albumin excretion (UAE), C-reactive protein (CRP), alanine aminotransferase (ALT) and γ-glutamyltransferase (γ-GT). In detail, the determination of blood lactate was performed by enzyme-electrode method (Biosen5030 Autocal glucose-lactate analyzer, EKF diagnostic Company, Germany) (22). Additionally, the calculation of the estimated glomerular filtration rate (eGFR) and the homeostasis model assessment of insulin resistance (HOMA2-IR) were described in our recent studies (19, 20).

### Diagnostic criteria

All T2DM subjects were diagnosed based on the WHO diagnostic criteria as our recent study (21). Based on the international expert consensus statement in 2020, the diagnosis of MAFLD in T2DM patients was consistent with our recent study (1, 23). The definition of obesity, smoking status and alcohol use referred to our recent studies (19, 20). Especially, mild obesity was defined as 25 kg/m^2^ ≤ BMI< 30 kg/m^2^, and severe obesity BMI ≥ 30 kg/m^2^. Mild abdominal obesity was defined as 90 cm ≤ WC < 100 cm in men and 80 cm ≤ WC < 90 cm in women, and severe abdominal obesity WC ≥ 100 cm in men and ≥ 90 cm in women.

### Statistical analysis

SPSS 15.0 software was used for statistical analysis. Data that conformed to a normal distribution were expressed as mean ± standard deviation, and the differences among multiple groups were determined by one-way analysis of variance (ANOVA) with the least significant difference (LSD). Whereas for non-normally distributed descriptive data, they were represented as medians with interquartile range (25-75%), and Kruskal Wallis test was applied for comparing the differences among multiple groups. Categorical variables were described as absolute numbers with percentages and were analyzed by the Chi-square test. When confounders were considered, categorical variables were corrected with binary logistic regression, and continuous variables were adjusted with univariate linear regression models. After non-normally distributed variables were transformed by normal score transformation, binary logistic regression was utilized to assess the associations of blood lactate levels and quartiles with the presence of MAFLD. *p <*0.05 (two-sided) was considered as statistically significant difference.

## Results

### Basal clinical characteristics of the subjects

**Table 1** demonstrates the general clinical characteristics of the T2DM subjects. The subjects were divided into quartiles based on blood lactate levels with the cutoffs of < 0.90, 0.90-1.15, 1.16-1.50, and > 1.50 mmol/l. The results presented that the patients in the higher lactate quartile were more likely to be females and older. After controlling for age and sex, the prevalence of hypertension and obesity, the percentages of the subjects taking LLD and metformin, WC, WHR, BMI, FPG, 2-h PPG, FCP, 2-h PCP, and TG progressively increased, whereas the percentages of the subjects taking IIAs gradually decreased from the lowest to the highest quartile (all *p* < 0.05). Additionally, the percentages of the smokers, HbA1c, HDL-C, LDL-C, and SUA were also significantly different among the four groups after adjustment for sex and age (all *p* < 0.05).

**Table 1:** Characteristics of the subjects according to quartiles.

Variables	Q1 (n=1152)	Q2 (n=1138)	Q3 (n=1174)	Q4 (n=1164)	*p* value	*p value
Blood lactate(mmol/l)	<0.90	0.90-1.15	1.16-1.50	>1.51	—	—
Male (n, %)^c^	680 (59.0%)	648 (56.9%)	621(52.9%)	470 (40.4%)	<0.001^γ^	<0.001^ϵ^
Age (years)^a^	59 ± 13	61 ± 12	61 ± 12	60 ± 12	0.012^α^	0.008^δ^
DD (months)^b^	84 (18-144)	84 (24-144)	84 (24-144)	84 (24-132)	0.993^β^	0.331^δ^
Hypertension (n, %)^c^	557 (48.4%)	609 (53.5%)	669 (57.0%)	675 (58.0%)	<0.001^γ^	<0.001^ϵ^
Obesity (n, %)^c^	416 (36.1%)	463 (40.7%)	538 (45.8%)	557 (47.9%)	<0.001^γ^	<0.001^ϵ^
Smoking (n, %)^c^	346 (30.0%)	327 (28.7%)	359 (30.6%)	247 (21.2%)	<0.001^γ^	0.028^ϵ^
Alcohol (n, %)^c^	187 (16.2%)	197 (17.3%)	177 (15.1%)	148 (12.7%)	0.016^γ^	0.410^ϵ^
IIAs (n, %)^c^	851 (73.9%)	822 (72.2%)	806 (68.7%)	747 (64.2%)	<0.001^γ^	<0.001^ϵ^
LLD (n, %)^c^	239 (20.8%)	310 (27.2%)	380 (32.4%)	485 (41.7%)	<0.001^γ^	<0.001^ϵ^
Metformin (n, %)^c^	592 (51.4%)	615 (54.0%)	690 (58.8%)	703 (60.4%)	<0.001^γ^	<0.001^ϵ^
Insulin sensitizers(n, %)^c^	98 (8.5%)	132 (11.6%)	120 (10.2%)	137 (11.8%)	0.038^γ^	0.054^ϵ^
SBP (mmHg)^a^	132 ± 18	133 ± 18	133 ± 18	133 ± 17	0.767^α^	0.936^δ^
DBP (mmHg)^a^	80 ± 10	80 ± 10	80 ± 10	80 ± 10	0.869^α^	0.610^δ^
WC (cm)^a^	87.71 ± 10.24	88.95 ± 10.10	90.09 ± 10.24	91.10 ± 9.86	<0.001^α^	<0.001^δ^
WHR^a^	0.91 ± 0.06	0.91 ± 0.07	0.92 ± 0.06	0.92 ± 0.06	<0.001^α^	<0.001^δ^
BMI (kg/m2)^a^	24.11 ± 3.44	24.49 ± 3.47	24.85 ± 3.38	25.08 ± 3.40	<0.001^α^	<0.001^δ^
FPG(mmol/l)^b^	7.79 (6.18-9.90)	7.86 (6.34-10.08)	7.88 (6.39-9.98)	8.17 (6.61-10.06)	0.030^β^	0.024^δ^
2-h PPG(mmol/l)^b^	13.39 (9.71-16.79)	13.79 (10.41-17.05)	13.91 (10.62-17.26)	14.26 (11.30-17.32)	<0.001^β^	<0.001^δ^
HbA1c (%)^a^	9.27 ± 2.50	9.31 ± 2.39	9.02 ± 2.23	8.80 ± 2.18	<0.001^α^	<0.001^δ^
FCP (ng/mL)^b^	1.53 (0.93-2.35)	1.69 (1.03-2.50)	1.85 (1.17-2.70)	2.07 (1.34-3.00)	<0.001^β^	<0.001^δ^
2-h PCP (ng/mL)^b^	3.25 (1.86-5.22)	3.67 (2.16-5.53)	4.09 (2.45-5.88)	4.89 (2.87-6.42)	<0.001^β^	<0.001^δ^
TG (mmol/l)^b^	1.21 (0.87-1.74)	1.34 (0.97-1.96)	1.51 (1.06-2.15)	1.85 (1.28-2.79)	<0.001^β^	<0.001^δ^
TC (mmol/l)^a^	4.64 ± 1.10	4.77 ± 1.18	4.76 ± 1.11	4.97 ± 1.09	<0.001^α^	0.086^δ^
HDL-C (mmol/l)^a^	1.15 ± 0.32	1.13 ± 0.33	1.11 ± 0.29	1.13 ± 0.30	0.014^α^	<0.001^δ^
LDL-C (mmol/l)^a^	2.99 ± 0.91	3.10 ± 0.91	3.06 ± 0.91	3.11 ± 0.89	0.007^α^	<0.001^δ^
Cr (μmol/l)^b^	67.0 (57.0-81.0)	67.0 (56.0-82.0)	67.0 (56.0-81.0)	65.0 (53.0-78.0)	<0.001^β^	0.603^δ^
SUA (μmol/l)^b^	298 (246-359)	294 (245-358)	317 (266-375)	325 (271-386)	<0.001^β^	<0.001^δ^
UAE (mg/24h)^b^	11.83 (6.70-30.56)	11.67 (6.71-28.63)	12.22 (7.06-32.95)	12.63 (7.21-36.19)	0.268^β^	0.414^δ^
eGFR (ml/min/1.73 m^2^)^b^	109.2 (88.9-131.1)	107.0 (87.3-130.7)	106.6 (86.8-130.1)	108.9 (90.3-132.4)	0.246^β^	0.688^δ^
CRP (mg/l)^b^	1.22 (0.49-3.48)	1.23 (0.54-3.39)	1.23 (0.59-3.48)	1.34 (0.57-3.26)	0.791^β^	0.997^δ^

a Data presented as mean ± S.D.

b Data presented as median with interquartile range.

c Data presented as numbers with percentages.

p^α^ value for comparison between groups, one-way ANOVA with the LSD.

p^β^ value for comparison between groups, the Kruskal-Wallis test.

p^γ^ value for comparison between groups, Chi-square test.

*p value: The p-values were adjusted for sex and age for the trend.

*p^δ^ value for comparison between groups, univariate linear regression.

*p^ϵ^ value for comparison between groups, binary logistic regression.

### Comparisons of MAFLD prevalence and blood lactate levels in different groups

**Figure 1:**
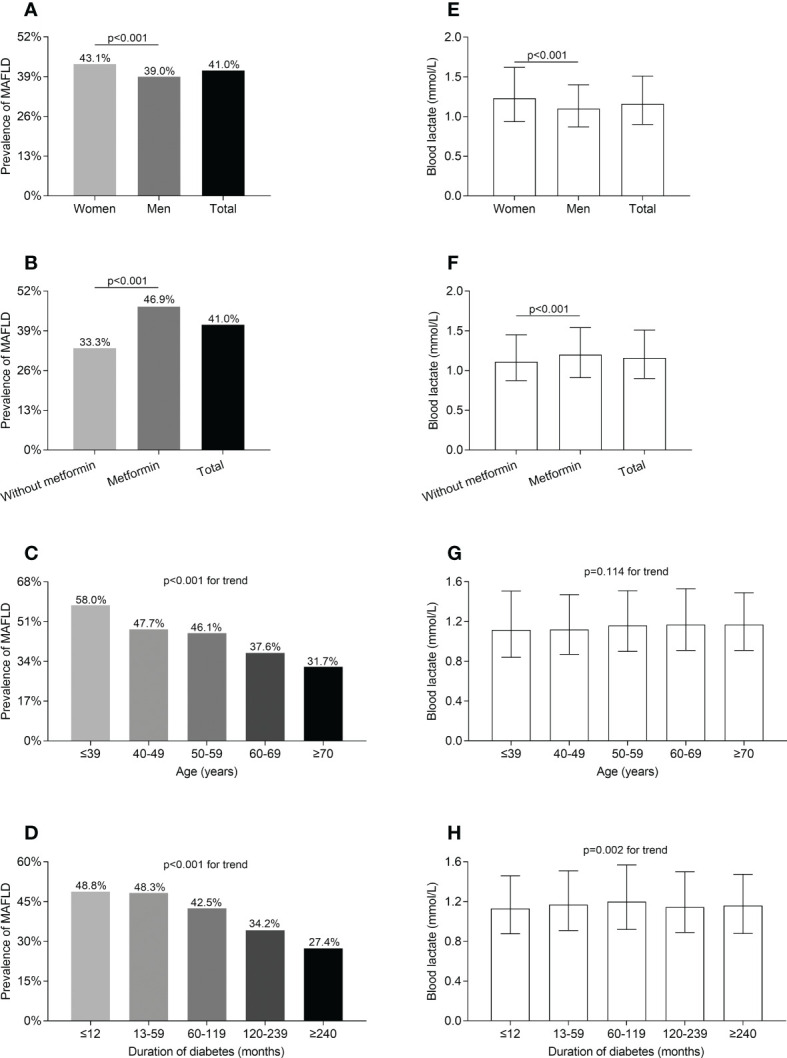
Comparisons of MAFLD prevalence and blood lactate level in T2DM patients stratified by sex, metformin use, age, and DD. **(A)** Comparison of MAFLD prevalence stratified by sex after adjusting for metformin use, age, and DD (*p*<0.001). **(B)** Comparison of MAFLD prevalence stratified by metformin use after adjusting for the sex, age and DD (*p*<0.001). **(C)** Comparison of MAFLD prevalence stratified by age after adjusting for sex, metformin use, and DD (*p*<0.001 for trend). **(D)** Comparison of MAFLD prevalence stratified by DD after adjusting for sex, metformin use, and age (*p*<0.001 for trend). **(E)** Comparison of blood lactate level stratified by sex after adjusting for metformin use, age, and DD (*p*<0.001). **(F)** Comparison of blood lactate level stratified by metformin use after adjusting for the sex, age, and DD (*p*<0.001). **(G)** Comparison of blood lactate level stratified by age after adjusting for sex, metformin use, and DD (*p*=0.114 for trend). **(H)** Comparison of blood lactate level stratified by DD after adjusting for sex, metformin use, and age (*p*=0.002 for trend).

**Figure 1** shows the comparisons of MAFLD prevalence and blood lactate levels in different T2DM groups. After adjustment for age, the use of metformin, and DD, the prevalence of MAFLD and blood lactate levels were obviously higher in women than in men (MAFLD prevalence: 43.1% vs 39.0%; blood lactate levels: 1.23 (0.94-1.62) mmol/L vs. 1.10 (0.87-1.40) mmol/L, respectively; all *p*<0.001) (**Figures 1A, E**). After adjustment for sex, age, and DD, the prevalence of MAFLD and blood lactate levels were significantly higher in the patients taking metformin compared with those without taking metformin (MAFLD prevalence: 46.9% vs 33.3%; blood lactate levels: 1.20 (0.91-1.54) mmol/L vs. 1.11 (0.87-1.45) mmol/L, respectively; all *p*<0.001) (**Figures 1B, F**). Moreover, the prevalence of MAFLD markedly decreased with increasing age and prolonging DD (all *p*<0.001 for trend) (**Figures 1C, D**). Additionally, the blood lactate levels were significantly different among the subjects stratified by DD after controlling for age, sex, and the use of metformin (*p*=0.002 for trend) (**Figure 1H**). However, after controlling for sex, the use of metformin and DD, there was no age-related significant difference in the blood lactate levels (**Figure 1G**).

### Comparisons of MAFLD prevalence across the blood lactate quartiles

**Figure 2:**
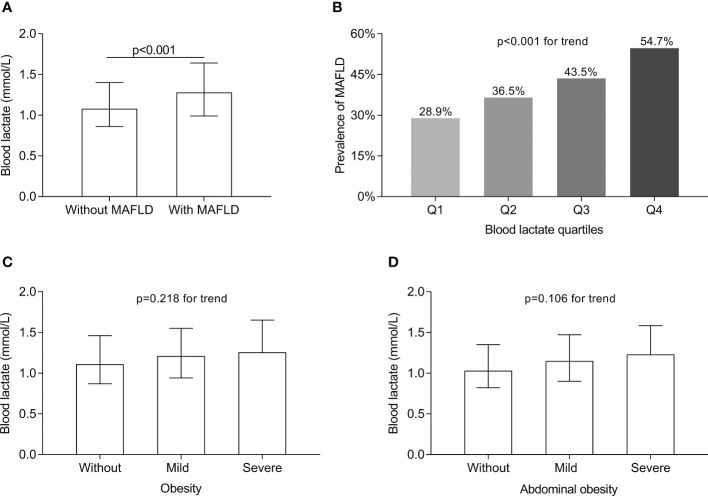
Comparisons of blood lactate level between the T2DM patients with and without MAFLD and MAFLD prevalence across the lactate quartiles. **(A)** Comparison of blood lactate level between the T2DM patients with and without MAFLD (*p*<0.001). **(B)** Comparison of MAFLD prevalence across the blood lactate quartiles (*p*<0.001 for trend). **(C)** Comparison of blood lactate level among the T2DM patients with different degree of obesity (*p*=0.218 for trend). **(D)** Comparison of blood lactate level among the T2DM patients with different degree of abdominal obesity (*p*=0.106 for trend).

**Figure 2A** displays the comparisons of blood lactate levels between the T2DM patients with and without MAFLD. The blood lactate levels in the T2DM patients with MAFLD was markedly higher (1.28 (0.99-1.64) mmol/L) than in those without MAFLD (1.08 (0.86-1.40) mmol/L) after controlling for age, sex, the use of metformin and DD (*p*<0.001) (**Figure 2A**). **Figure 2B** shows the comparisons of MAFLD prevalence across the blood lactate quartiles. A significantly increased trend in the prevalence of MAFLD from the lowest to the highest lactate quartiles in the T2DM patients after adjustment for age, sex, the use of metformin and DD was observed (28.9%, 36.5%, 43.5%, and 54.7% for the first, second, third, and fourth quartiles, respectively, *p*<0.001 for trend) (**Figure 2B**). **Figure 2C** shows the comparisons of blood lactate levels between the T2DM patients with different degree of obesity. After controlling for age, sex, the use of metformin and DD, there was no obesity-related significant difference in the blood lactate levels (*p*=0.218 for trend) (**Figure 2C**). **Figure 2D** shows the comparisons of blood lactate levels between the T2DM patients with different degree of abdominal obesity. After controlling for age, sex, the use of metformin and DD, there was also no abdominal obesity-related significant difference in the blood lactate levels (*p=*0.106 for trend) (**Figure 2D**).

### Comparisons of serum ALT and γ-GT levels

**Figure 3:**
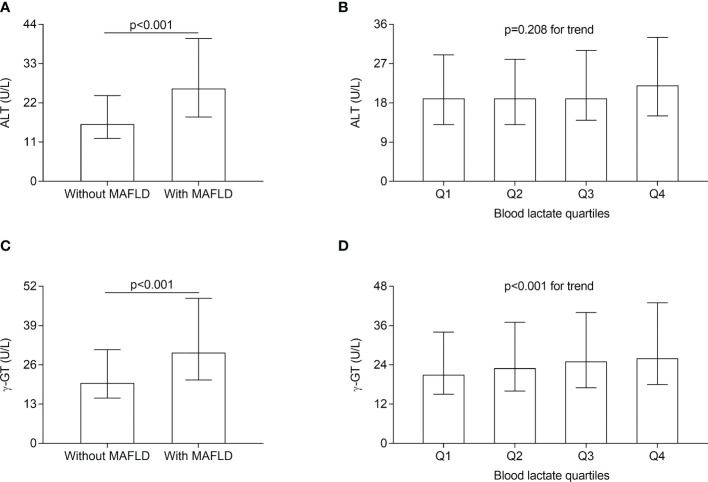
Comparisons of serum ALT and γ-GT level in different groups. **(A)** Comparisons of serum ALT level between the T2DM patients with and without MAFLD (*p* < 0.001). **(B)** Comparisons of serum ALT level across the blood lactate quartile (*p*= 0.208 for trend). **(C)** Comparisons of serum γ-GT level between the T2DM patients with and without MAFLD (*p* < 0.001). **(D)** Comparisons of serum γ-GT levels across the blood lactate quartile (*p* < 0.001 for trend).

The comparisons of serum ALT and γ-GT levels in different groups are presented in **Figure 3**. After correcting for age, sex, the use of metformin, and DD, both serum ALT and γ-GT levels were significantly higher in the T2DM patients with MAFLD compared with those without MAFLD (serum ALT levels: 26 (18-40) vs. 16 (12-24) U/L; serum γ-GT levels: 30 (21-48) vs. 20 (15-31) U/L respectively; all *p*<0.001) (**Figures 3A, C**). Furthermore, after adjusting for sex, age, the use of metformin and DD, serum γ-GT levels significantly rose with the increasing lactate quartiles in the T2DM patients (21 (15-34), 23 (16-37), 25 (17-40), 26 (18-43) U/L for the first, second, third, and fourth quartiles, respectively, *p*<0.001 for trend) (**Figure 3D**). Whereas serum ALT levels were not significantly different among the lactate quartiles (**Figure 3B**).

### Correlations of blood lactate levels with insulin resistance

**Figure 4:**
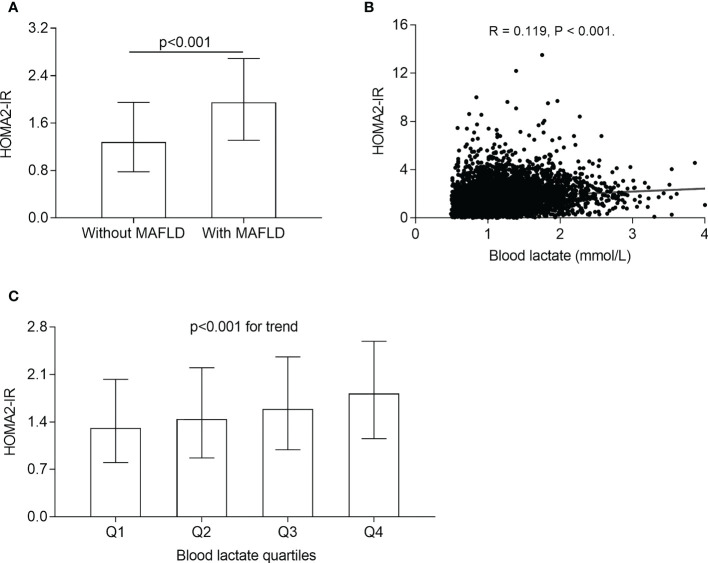
Correlation of blood lactate with insulin resistance. **(A)** Comparison of HOMA2-IR between the T2DM patients with and without MAFLD (*p*<0.001). **(B)** Correlation of blood lactate level with HOMA2-IR after adjusting for sex, age, metformin use, and DD (*p*<0.001). **(C)** Comparison of HOMA2-IR across the blood lactate quartile (*p*<0.001 for trend).

The comparisons of HOMA2-IR in different groups and the correlation of blood lactate levels with HOMA2-IR are illustrated in **Figure 4**. After adjustment for sex, age, the use of metformin and DD, the HOMA2-IR were obviously higher in the T2DM patients with MAFLD (1.95 (1.31-2.69)) than those without MAFLD (1.28 (0.78-1.95)) (*p*<0.001) (**Figure 4A**). The significantly increased trend in HOMA2-IR were observed across the blood lactate quartiles (1.31 (0.80-2.03), 1.44 (0.87-2.20), 1.59 (0.99-2.36), 1.82 (1.15-2.59) for the first, second, third, and fourth quartiles, respectively, *p*<0.001 for trend) (**Figure 4C**). In addition, partial correlation analysis revealed that the blood lactate was positively corelated with HOMA2-IR in T2DM patients (R=0.119, *p*<0.001) (**Figure 4B**).

### Association of blood lactate levels with MAFLD

**Table 2** demonstrates the association of blood lactate levels with the presence of MAFLD in T2DM patients stratified by metformin use. Logistic regression showed that before (Model 1) and after correcting for age, sex, and DD (Model 2), blood lactate levels were significantly associated with the presence of MAFLD in both T2DM patients with (Model 1: OR=1.525, 95%CI: 1.404-1.658; Model 2: OR=1.566, 95%CI: 1.438-1.706, respectively, all *p*<0.001) and without taking metformin (Model 1: OR=1.482, 95%CI: 1.344-1.635; Model 2: OR=1.451, 95%CI: 1.312-1.605, respectively, all *p*<0.001). After further adjusting for obesity, smoking status, and alcohol drinking (Model 3), use of LLD, IIAs, and insulin sensitizers (Model 4), anthropometric indices including WC, WHR, and BMI (Model 5), and laboratory parameters including TC, HDL-C, LDL-C, TG, eGFR, SUA, UAE, FPG, 2-h PPG, HbA1c, CRP, FCP, and 2-h PCP (Model 6), the significantly positive correlation between blood lactate levels and the presence of MAFLD still existed in both T2DM patients with (Model 3: OR=1.570, 95%CI: 1.434-1.719; Model 4: OR=1.519, 95%CI: 1.386-1.665; Model 5: OR=1.506, 95%CI: 1.341-1.692; Model 6: OR=1.378, 95%CI: 1.210-1.569, respectively, all *p*<0.001) and without taking metformin (Model 3: OR=1.406, 95%CI: 1.263-1.565, *p*<0.001; Model 4: OR=1.335, 95%CI: 1.197-1.488, *p*<0.001; Model 5: OR=1.311, 95%CI: 1.144-1.501, *p*<0.001; Model 6: OR=1.181, 95%CI: 1.010-1.381, *p*=0.037, respectively).

**Table 2:** Association of blood lactate with the prevalence of MAFLD.

	B statistic	OR	95% CI	*p* value
Without metformin				
Model 1	0.394	1.482	1.344-1.635	<0.001
Model 2	0.372	1.451	1.312-1.605	<0.001
Model 3	0.341	1.406	1.263-1.565	<0.001
Model 4	0.289	1.335	1.197-1.488	<0.001
Model 5	0.270	1.311	1.144-1.501	<0.001
Model 6	0.166	1.181	1.010-1.381	0.037
Metformin				
Model 1	0.422	1.525	1.404-1.658	<0.001
Model 2	0.449	1.566	1.438-1.706	<0.001
Model 3	0.451	1.570	1.434-1.719	<0.001
Model 4	0.418	1.519	1.386-1.665	<0.001
Model 5	0.410	1.506	1.341-1.692	<0.001
Model 6	0.320	1.378	1.210-1.569	<0.001

Model 1: unadjusted.

Model 2: Adjusted for age, sex, and DD.

Model 3: Further adjustment for obesity, smoking status, and alcohol drinking.

Model 4: Further adjustment for use of LLD, IIAs, and insulin sensitizers.

Model 5: Further adjustment for WC, WHR, and BMI.

Model 6: Further adjustment for TC, HDL-C, LDL-C, TG, eGFR, SUA, UAE, FPG, 2-h PPG, HbA1c, CRP, FCP, and 2-h PCP.

### Association of lactate quartiles with the prevalence of MAFLD

**Table 3** presents the association of blood lactate quartiles with the presence of MAFLD in T2DM patients. Before (Model 1) and after adjustment for age, sex, and DD (Model 2), the subjects in higher lactate quartiles showed a significantly higher risk for MAFLD (*p*<0.001 for trend). After further controlling for obesity, smoking status, and alcohol drinking (Model 3), medication usage (Model 4), physical measurements (Model 5), and laboratory examinations results (Model 6), higher lactate quartiles remained significantly correlated to an increased risk for MAFLD (all *p*<0.001 for trend). Compared with the subjects in the lowest blood lactate quartiles, the risk of MAFLD increased to 1.436-, 1.473-, and 2.055-fold, respectively, in those from the second to the highest lactate quartiles.

**Table 3:** Association of blood lactate quartiles with the prevalence of MAFLD.

	ORs (95% CI)				*p* values for trend
	Q1	Q2	Q3	Q4	
Model 1	1	1.412 (1.185-1.683)	1.896 (1.596-2.251)	2.973 (2.503-3.530)	<0.001
Model 2	1	1.475 (1.232-1.765)	1.989 (1.668-2.373)	3.052 (2.556-3.645)	<0.001
Model 3	1	1.455 (1.201-1.764)	1.828 (1.514-2.208)	2.966 (2.453-3.586)	<0.001
Model 4	1	1.391 (1.146-1.688)	1.713 (1.415-2.073)	2.643 (2.179-3.205)	<0.001
Model 5	1	1.444 (1.140-1.829)	1.614 (1.275-2.043)	2.683 (2.105-3.421)	<0.001
Model 6	1	1.436 (1.102-1.870)	1.473 (1.129-1.922)	2.055 (1.563-2.702)	<0.001

Model 1: unadjusted.

Model 2: Adjusted for age, sex, and DD.

Model 3: Further adjustment for obesity, smoking status, and alcohol drinking.

Model 4: Further adjustment for use of LLD, IIAs, and insulin sensitizers.

Model 5: Further adjustment for WC, WHR, and BMI.

Model 6: Further adjustment for TC, HDL-C, LDL-C, TG, eGFR, SUA, UAE, FPG, 2-h PPG, HbA1c, CRP, FCP, and 2-h PCP.

## Discussion

It’s well known that T2DM is closely related to the development and progression of MAFLD, a hepatic manifestation of multisystem metabolic dysfunction. Notably, a meta-analysis demonstrated that MAFLD was more prevalent in T2DM patients (51.83%) than in non-diabetics (30.76%) (7). In the present real-world study, the prevalence of MAFLD in T2DM inpatients was 41.0%, which was close to the recent findings reported by our team (41.2%) (23).

The close link between T2DM and MAFLD is attributed to their common pathogenic mechanism and metabolic risk factors such as genetic factors, unhealthy lifestyle, insulin resistance, and abnormal lipid metabolism (24). More importantly, the presence of T2DM further exacerbates the progression of MAFLD and the patients with type 2 diabetes are at a higher risk of hepatic fibrosis and cirrhosis (25, 26). Furthermore, the coexistence of T2DM and MAFLD increased the risk of developing end-stage hepatocellular carcinoma and aggravated the progression of both macro- and microvascular complications of diabetes (27, 28). Thus, early prediction and assessment of the risk of MAFLD and timely intervention are of great significance in T2DM patients.

As the end product of anaerobic glycolysis and an essential energy substance that is transferred within and between tissues, circulating lactate is the primary carbon source of the tricarboxylic acid cycle in anaerobic condition and plays an important role in the overall energy metabolism and signal transduction of living organisms (10, 29, 30). Lactate is mainly produced in skeletal muscle, released into the circulation and metabolized back to glucose in liver and kidney *via* gluconeogenesis, serving as an energy carrier for reuse in various organs, which constitutes the Cori cycle (31). Additionally, liver is also the major organ closely associated with lactate removal. Therefore, liver plays an important role in the metabolism of lactate and thus there may exist an interacting association between blood lactate and liver diseases including MAFLD (15, 16, 32).

Interestingly, several previous studies displayed that blood lactate may be an indicator of hepatocellular failure and the increased lactate levels were observed in some liver diseases (15, 16, 32). For instance, Johanne et al. found that blood lactate levels seem to increase with the severity of liver diseases, especially in cirrhosis (16). Additionally, Ferriero et al. observed that an elevated lactate concentration in liver nuclear fraction induced expression of damage response gene, which further aggravated liver injury (32). Nevertheless, the close association of blood lactate with MAFLD was observed only in a few studies (11–13), and few in population especially in T2DM population. For example, an analysis of serum metabolites showed that serum lactate levels were significantly elevated in the diet-induced mice model of NAFLD, which was also present in NAFLD patients with steatosis alone (12). Additionally, plasma lactate levels were confirmed to be higher in non-diabetic patients with hepatic steatosis and steatohepatitis compared with normal healthy controls (13).

Therefore, we conducted the present study to investigate the real relationship between blood lactate levels and MAFLD in T2DM subjects. Our present study showed that blood lactate levels were significantly higher in women patients than in men patients, which corresponded to the distribution of the prevalence of MAFLD stratified by sex. Since metformin promotes anaerobic glycolysis and thus increases blood lactate levels, we observed that blood lactate levels were significantly higher in the patients taking metformin than in those without taking metformin in our study. More importantly, compared with T2DM patients without MAFLD, the blood lactate levels were significantly elevated in those with MAFLD, and the MAFLD prevalence obviously increased with increasing lactate levels.

It’s worth noting that many factors such as heredity, diet structure, and lifestyle are closely associated with MAFLD, which also influence lactate metabolism and levels (33–42). For example, by giving a conventional diet first and following a low-carbohydrate diet for four weeks, Michalczyk el al. conducted a dietary intervention experiment in 15 competitive basketball players to observe the change in blood lactate levels. They found that the low-carbohydrate diet procedure significantly reduced the blood lactate concentrations of the subjects (39). Interestingly, high-carbohydrate diets were significantly associated with an increased risk of MAFLD (35). Additionally, regular physical training can increase lactate clearance during rest and result in decreased blood lactate levels (40), while aerobic exercise intervention has a potential benefit in improving the histological endpoint of MAFLD (37, 38). Therefore, some factors such as diet and exercise can influence the development of MAFLD and the metabolism of lactate, but their specific effects on the relationship between lactate and MAFLD are complex and not yet fully understood, which needs to be further explored.

Furthermore, a significantly positive correlation between blood lactate levels and the presence of MAFLD were observed in T2DM patients with and without taking metformin even after adjustment for other confounding factors such as obesity and dyslipidemia. Higher lactate quartiles showed a significantly higher risk for MAFLD. The MAFLD prevalence of the subjects in the fourth blood lactate quartile (blood lactate >1.51 mmol/l) increased almost 2-fold compared with those in the first blood lactate quartile (blood lactate <0.90 mmol/l). Given that metformin can increase blood lactate levels (18), therefore we further divided the subjects into two groups including the patients with and without metformin therapy to exclude the effect of metformin. Even so, blood lactate remained independently associated with the presence of MAFLD in both the patients taking and not taking metformin.

Notably, lactate not only responds to the presence of MAFLD, but also to the extent and severity of fatty liver. We found the serum γ-GT levels, one of sensitively enzyme indicators for diagnosis of liver injury and MAFLD, significantly rose with the increasing lactate quartiles in the T2DM patients, whereas there was no difference in serum ALT levels across the lactate quartiles. Slightly contrary to our results, previous study illustrated that serum lactate levels positively correlated with serum ALT but not γ-GT levels in T2DM patients (43). Because both serum ALT and γ-GT are indicators to evaluate the severity of liver injury, all the above studies including ours suggest that blood lactate levels assess not only the risk of developing MAFLD, but also the severity of MAFLD.

The close association between blood lactate and MAFLD may attribute to insulin resistance induced by blood lactate. As an underlying pathophysiology of MAFLD, insulin resistance inhibits β-oxidation of free fatty acids; increases the flow of free fatty acids from adipose tissue to liver; and upregulates hepatic lipogenic transcription factors that stimulates hepatic *de novo* lipogenesis, which lead to hepatic steatosis and subsequent the development and progression of MAFLD (44, 45). Accordingly, our findings indicated that the patients with higher blood lactate levels accompanied with a severer insulin resistance assessed by HOMA2-IR and blood lactate level was positively related to HOMA2-IR in T2DM patients. Some previous studies supported our viewpoints (46–48). For example, a longitudinal study implicated that insulin resistance was associated with higher concentration of serum lactate in healthy children after analyzing serum metabolites, and more importantly, elevated lactate levels preceded the increased levels of insulin resistance (46). Therefore, the accumulation of lactate in adipocytes is a key trigger mediating systemic insulin resistance and in turn, hyperinsulinemia participates in glycolysis and enhances the Warburg effect, resulting in the production of large amounts of lactate (47, 48).

Additionally, the increase of blood lactate in MAFLD maybe the result of decreased lactate clearance due to liver dysfunction caused by MAFLD (18). A previous study mentioned that endogenous lactate was cleared from the bloodstream almost three times more slowly in hepatic cirrhotic patients than in healthy individuals (49). Wang et al. also observed impaired hepatic lactate clearance in NAFLD mouse model fed with high fat diet, which in turn led to lactate accumulation (14). Additionally, the T2DM subjects with high lactate levels were usually accompanied by more risk factors associated with MAFLD in the present study. For example, the participants in higher lactate quartile were more likely to develop metabolic disturbance such as dyslipidemia, hypertension, and obesity, which might accelerate the development of MAFLD in T2DM.

However, some limitations must be mentioned. Firstly, our study is a cross-sectional study and thus the causal relationship between blood lactate and MAFLD in T2DM patients cannot be clarified and the change of blood lactate levels with the improve or deteriorate of MAFLD also cannot be tracked. Secondly, subjects recruited in this study were from single-center. Therefore, a large, multi-center, prospective study is needed to extend the study subjects to not only T2DM population but also other populations to determine the applicability of blood lactate to assess the risk of MAFLD. Thirdly, the diagnosis of MAFLD in this study was based on ultrasonography, whereas the gold standard for MAFLD diagnosis is liver biopsy, and ultrasound diagnosis tends to miss a proportion of patients with the degree of steatosis less than 30% (50); but even so, ultrasonography is still the recommended first-line, non-invasive, and reliable diagnostic modality to screen for the presence of liver steatosis in clinical setting, especially in large-scale population studies (1, 51). Finally, our study lacked quantitative data on MAFLD to determine the degree of severity, such as fibrosis score and degree of steatosis assessed by liver transient elastography. However, it has been observed that serum ALT and γ-GT levels increased with the degree of fibrosis determined by transient elastography (52, 53), so in the present study, it is feasible to use serum ALT and γ-GT levels to indicate the severity of MAFLD.

In conclusion, the present study with a relatively large sample provided novel clinical evidence to illustrate the independent association between blood lactate and the increased risk of MAFLD in type 2 diabetes regardless of metformin use, which may closely correlate with insulin resistance. Blood lactate levels might be used as a practical indicator for assessing the risk and severity of MAFLD in T2DM patients.

## Data availability statement

The original contributions presented in the study are included in the article/supplementary material. Further inquiries can be directed to the corresponding author.

## Ethics statement

The studies involving human participants were reviewed and approved by the ethics committee of Shanghai Sixth People’s Hospital Affiliated to Shanghai Jiao Tong University School of Medicine. The patients/participants provided their written informed consent to participate in this study.

## Author contributions

L-XL designed the study, reviewed, and edited the manuscript. Y-LM, Y-JW, and M-RX collected samples and clinical data. Y-LM, J-FK and J-WW worked together, performed statistical analysis, and wrote the manuscript. All authors contributed to the article and approved the submitted version.
